# Implementing and Evaluating the Impact of BoneRx: A Healthy Bone Prescription for Men with Prostate Cancer Initiating Androgen Deprivation Therapy

**DOI:** 10.3390/jcm11102703

**Published:** 2022-05-11

**Authors:** Jennifer M. Jones, Derek S. Tsang, Shiyu Zheng, Ariel Yeheskel, Charles N. Catton, Angela M. Cheung, Robert Hamilton, Shabbir M. H. Alibhai

**Affiliations:** 1 Cancer Rehabilitation and Survivorship Program, Department of Supportive Care, Princess Margaret Cancer Centre, 200 Elizabeth Street, Toronto, ON M5G 2C4, Canada; s.zheng@queensu.ca; 2 Raditation Medicine Program, Princess Margaret Cancer Centre, 610 University Av., Toronto, ON M5G 2M9, Canada; derek.tsang@rmp.uhn.ca (D.S.T.); charles.catton@rmp.uhn.ca (C.N.C.); 3 Faculty of Medicine, University of Toronto, 1 King’s College Cir, Toronto, ON M5S 1A8, Canada; ariel.yeheskel2@uhn.ca; 4 Osteoporosis Program and Centre of Excellence in Skeletal Health Assessment, University Health Network, 200 Elizabeth Street, 7EN-Rm 221, Toronto, ON M5G 2C4, Canada; angela.cheung@uhn.ca; 5 Department of Surgery (Urology), University of Toronto, 610 University Av., Toronto, ON M5G 2M9, Canada; rob.hamilton@uhn.ca; 6 Department of Medicine, University Health Network, 200 Elizabeth Street, Toronto, ON M5G 2C4, Canada; shabbir.alibhai@uhn.ca

**Keywords:** prostate cancer, androgen deprivation therapy, osteoporosis, bone health, patient education

## Abstract

Background: The initiation of Androgen Deprivation Therapy (ADT) results in rapid and profound hypogonadism, resulting in significant bone and muscle loss, increasing the risk for osteoporosis (OP), falls, and fractures. Despite this, there exist very low rates of guideline adherent care regarding bone health in this population. We developed and implemented a healthy bone prescription tool entitled BoneRx to facilitate the uptake of guideline-concordant bone health care into practice and increase patient awareness and promote the uptake of health bone behaviours (HBBs). Methods: We conducted a cross-sectional pre-BoneRx implementation (*n* = 143) vs. post-implementation (*n* = 149) cohort study to evaluate the impact on (i) bone health care, patient engagement in HBB, and patient knowledge and health beliefs regarding OP. Results: There was a significant difference pre- vs. post BoneRx implementation on receipt of baseline BMD (34.7% vs. 59.5%, *p* < 0.0001) and bone health counselling (32.4% vs. 59.9%, *p* < 0.0001). More participants in the post-BoneRx implementation cohort reported taking vitamin D supplements 57% vs. 81% (*p* < 0.001) and calcium supplements 39% vs. 61% (*p* < 0.001). Physical activity levels also significantly increased (*p* = 0.021). No differences were detected in OP knowledge or feelings of OP susceptibility, seriousness, or health motivation. Conclusion: BoneRx is a simple, cost-effective, and acceptable strategy that could improve the care of PCa survivors receiving ADT.

## 1. Introduction

Androgen deprivation therapy (ADT) is an effective and increasingly common treatment for men with prostate cancer (PCa) [1,2]. Almost half of men diagnosed with prostate cancer are now expected to receive this treatment [1,2,3] and may remain on it for up to two decades [4]. While ADT has been shown to reduce tumour growth and disease-specific symptoms and extend survival [5,6,7,8,9], it is also associated with adverse effects including bone loss [10,11,12,13,14].

The initiation of ADT results in rapid and profound hypogonadism, resulting in significant bone and muscle loss, increasing the risk for osteoporosis (OP), falls, and fractures [14,15,16]. Men receiving ADT have been shown to have a 5- to 10-fold increased loss of bone mass [14,17,18], and the risk of fracture has been reported up to 20% by 5 years of treatment [19,20,21,22], an at least 50% greater risk of fractures than in healthy controls or men with PCa who are not on ADT [20,21,22,23]. In a large SEER database study, 58% of men at high risk and 38% of men at low risk for fracture at baseline developed at least one fracture after ADT [24]. Fractures secondary to OP can result in severe pain, fatigue, depression, and functional impairment [25,26]. In men with prostate cancer, fractures are associated with up to 40% excess mortality [24,27].

Guidelines and consensus statements recommend the use of baseline screening and routine follow-up bone mineral density (BMD), prophylactic pharmacologic therapy for those at high risk for fracture, and in some cases referral to bone health specialists/programs [28,29,30,31,32]. In addition, guidelines recommend that men initiating ADT should receive education regarding cancer treatment-induced bone loss and should be educated on the initiation and maintenance of healthy bone behaviours (HBBs), including exercise, optimizing vitamin D, and calcium intake [28,33,34,35,36].

However, research from our team and others have consistently demonstrated very low rates of guideline adherent care regarding bone health in this population [37,38,39,40,41]. In a large study of Veterans Health Administration data (*n* = 17,017), only 15–20% of men with prostate cancer who received any ADT between 2005 and 2014 received a BMD test within a three-year period of ADT initiation [41]. In the same study, those men who received a BMD test were more likely to have received osteoporosis and fracture diagnoses, use of vitamin D, calcium, and bisphosphonates. Gaps in PCa specialists’ knowledge regarding bone health have been reported [42,43] and the majority of men on ADT are unaware that bone loss is a side effect of ADT, have low knowledge about OP, and are not engaging regularly in HBBs, particularly calcium and vitamin D intake and exercise [44,45,46]. Barriers to implementing guidelines related to bone health, which include lack of time and supporting structures (i.e., tools and patient education materials), have been reported [43].

These findings suggest an urgent need to address this knowledge to practice gap and to develop simple cost-effective tools that target both PCa health care practitioners (HCPs) and patients and facilitate the communication of clear recommendations, prompt guideline-adherent practice, as well as provide patient education to increase awareness and promote HBBs. Previous systematic reviews of both point-of-care reminders and patient-mediated interventions have reported small to moderate increases in outcomes related to adherence to clinical recommendations and patient outcomes [47,48,49]. Further, interventions that target HCPs and patients and that include both reminders and education have been shown to be most effective in bridging the gap between evidence and clinical practice for OP [50]. In response, we developed a healthy bone prescription tool that was guided by a theory-based knowledge use framework, entitled BoneRx. The goal of BoneRx is to facilitate the uptake of guideline-concordant bone health care into practice and increase patient awareness and promote the uptake of HBBs.

The specific aims of the study were to implement BoneRx in a busy prostate clinic and evaluate the impact of BoneRX on (i) bone health care (BMD ordering, counselling), (ii) patient engagement in HBBs, and (iii) patient knowledge and health beliefs regarding OP. Further, we assessed patient satisfaction with the BoneRx intervention (post-implementation cohort).

## 2. Materials and Methods

We conducted a cross-sectional pre-implementation vs. post-implementation cohort study to assess the impact of BoneRx. This study was conducted at Princess Margaret Cancer Centre (PM), the largest single-site cancer hospital in Canada. The Prostate Centre at PM treats over 1000 men with PCa each year, of which approximately 20–30% are prescribed ADT. Ethics approval was obtained through the University Health Network Research Ethics Board and participants provided informed written consent.

### 2.1. BoneRx Intervention

**Figure 1:**
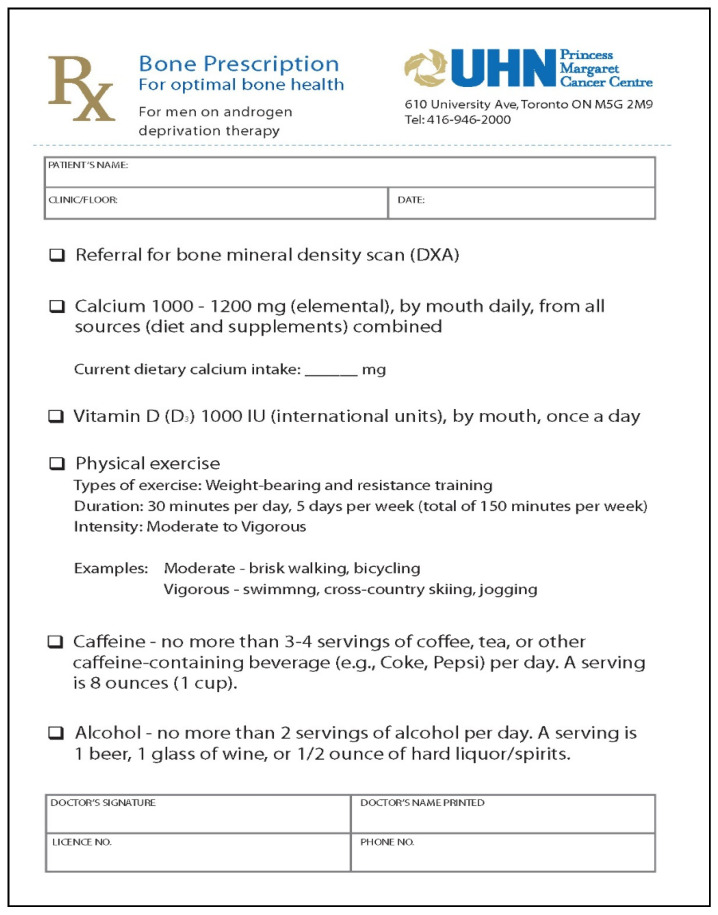
BoneRx Healthy Bone Prescription.

The intervention entitled BoneRx was provided to PCa patients at the initiation of ADT and served as both a reminder/prompt for guideline-adherent practice for the PCa HCP and targeted education for the patient. BoneRx consists of two elements: (1) a pre-populated “healthy bones prescription” (see Figure 1) which prompts the PCa specialist to order a BMD test and includes clear guideline-specific recommendations to the patient in terms of calcium (1000–1200 mg/daily through diet and/or supplementation) and vitamin D intake (1000 IU/daily through supplement) and physical activity (working towards 150 min of moderate-to-vigorous physical activity/week); and (2) a patient booklet entitled “Building Strong Bones: For Men Taking Androgen Deprivation Therapy.” This booklet, which was developed and pilot tested at PM in collaboration with OP, PCa and patient education specialists, provides clear information on the effects of ADT on bone, information about HBB guidelines, pictures of calcium-rich foods, suggestions for implementing HBBs, types/brands of vitamin D supplementation, and links to further reliable resources [51] (available upon request).

To facilitate the implementation of BoneRx into the PM prostate clinic, we employed multiple enabling and reinforcing strategies based on the Awareness-to-Adherence model of behaviour change [52,53]. The preliminary consultation, diffusion, and dissemination strategy included the following strategies to promote *awareness* and *agreement*: (1) Gathering feedback from stakeholders (including HCPs and patients) on intervention materials and workflow to fine-tune the implementation approach; (2) Presentations to the site teams at weekly tumour boards/rounds (and copy via email) to increase awareness, target attitudes, and provide an introduction to the BoneRx tools and workflow. Following final revisions to the implementation approach, BoneRx was rolled out in the PM Prostate Clinic. Following initial implementation (3 months), we employed the following strategies to facilitate *adoption* and *adherence*: (1) Audit and feedback were conducted in each clinic to document if BoneRx had been provided and documented with the target population; (2) Reminders regarding the BoneRx intervention were integrated into routine clinical care team meetings/rounds and sent via e-mail to PCa HCPs as part of the stimulus to the change in practice expected; (3) Information posters were developed to remind PCa HCPs to use the BoneRx tool and to inform patients about BoneRx.

### 2.2. Procedure and Participants

**Figure 2:**
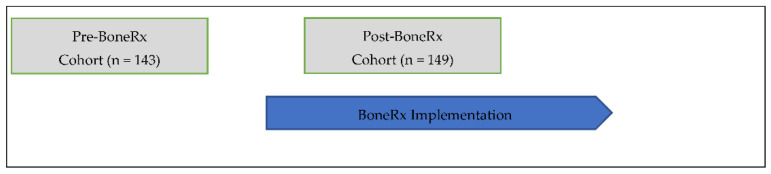
Study design.

At pre-implementation and post implementation, eligible patients were approached during their regularly scheduled six-month post-ADT initiation appointment and asked if they would be willing to complete a questionnaire package. Participants had to be able to understand English and provide informed consent. They were excluded if they were receiving concurrent chemotherapy or had symptomatic metastatic disease. The post-implementation cohort received the BoneRx intervention at ADT initiation (as part of standard of care). The pre-implementation cohort group did not receive the BoneRx intervention. Chart audit was conducted for both cohort groups (see Figure 2).

### 2.3. Outcome Measures

Using a standardized data extraction form, chart audits were conducted and included: (1) date of diagnosis and current treatment details; (2) any history of BMD test; (3) BMD ordered within 6 months of ADT initiation.

Participants completed a questionnaire package which included the following sections: (1) Demographics and osteoporosis risk factors (including height, weight, fracture and fall history, steroid use, and tobacco and alcohol use); (2) Bone health knowledge assessed using the 19-item revised Facts on Osteoporosis Quiz-Revised [54], which included the 5-item Men’s Osteoporosis Knowledge Questionnaire [55]; (3Health beliefs regarding bone health assessed using three subscales (susceptibility, seriousness and health motivation) of the Osteoporosis Health Belief Scale [56], and (4) Healthy bone behaviours which included use of calcium and vitamin D supplementation, calcium intake (diet), and physical activity [44,57,58,59]. In the post-BoneRx implementation cohort group, 8 questions were added to gather feedback on the participants’ experience of receiving BoneRx.

### 2.4. Statistical Analyses

Data were examined for normality and are presented as mean (SD), median (range), or number (percentage) as appropriate. Statistical comparisons between the pre and post-BoneRX implementation cohort groups were performed using independent t tests, Mann-Whitney U tests or Pearson’s chi-square tests, respectively. Statistical analyses were performed by IBM SPSS Statistics, version 21 (IBM, Armonk, New York, NY, USA). Two-tailed *p* values of <0.05 were considered statistically significant.

## 3. Results

There were 143 participants in the pre-implementation cohort (72% recruitment rate) and 149 participants in the post-intervention cohort (86% recruitment rate). The cohorts did not differ on demographic or clinical variables, with the exception of education and falls in the past 12 months (see Table 1).

### 3.1. Bone Health Care

**Figure 3:**
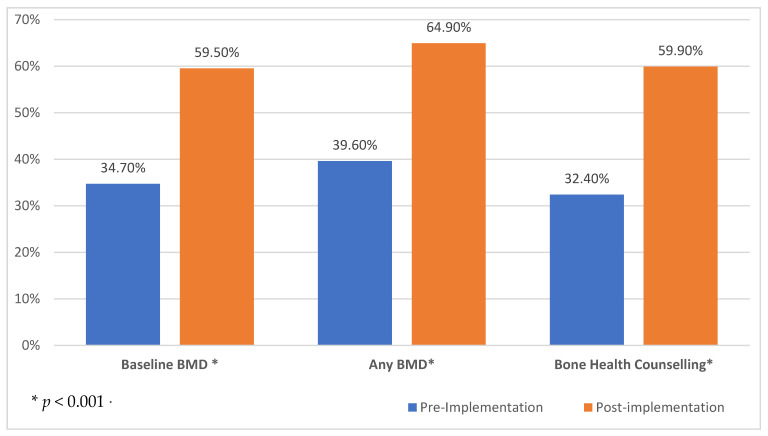
Baseline Bone Mineral Density (BMD) Test.

The number of PCa survivors who underwent a BMD test within 6 months of ADT initiation was significantly different from 34.7% at pre-BoneRx implementation to 59.5% at post-BoneRx implementation (*p* < 0.0001) (see Figure 3). The record of any BMD test in the chart also differed between the cohorts (pre 39.6% vs. post 64.9%, *p* < 0.001). The proportion of patients who reported that they had received counselling about bone health was 32.4% in the pre-implementation cohort and 59.9% in the post implementation cohort (*p* < 0.0001) (see Figure 3).

### 3.2. Healthy Bone Behaviours

Significantly more participants in the post-BoneRx implementation cohort reported taking vitamin D supplements 57% vs. 81% (*p* < 0.001). The mean daily calcium intake through diet did not significantly differ between the two cohorts (779 mg + 413 vs. 736 mg + 499, *p* = 0.44). However, significantly more participants were taking calcium supplements between pre- (39%) and post- (61%) implementation (*p* < 0.001). Furthermore, the proportion of men who were not meeting the recommended amount of calcium (1000–1200 mg) through their diet and were also not taking calcium supplements decreased from 42% in the pre- vs. to 29% post-BoneRx implementation cohort (*p* = 0.027).

**Figure 4:**
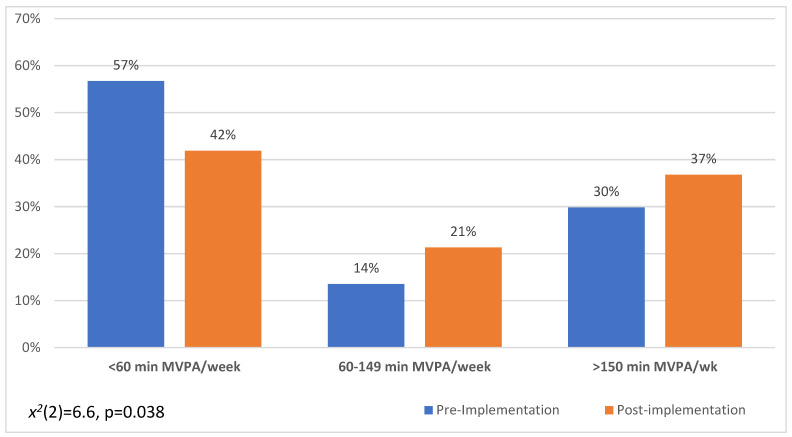
Moderate-to-Vigorous Physical Activity (MVPA).

The median number (skewed data) of minutes per week that participants engaged in moderate to vigorous physical activity (MVPA) was significantly lower (median = 0) in the pre-implementation cohort compared to the post-implementation cohort (median = 95 min) (*p* = 0.021). Furthermore, the proportion of participants who engaged in some (60–149 min/week) or guideline adherent levels (>150 min week) of MVPA was also significantly higher in the post-intervention cohort (*p* = 0.038) (see Figure 4).

### 3.3. Osteoporosis Knowledge and Health Beliefs

There was no difference in the OP knowledge scores or feelings of OP susceptibility, seriousness, or health motivation between the pre- and post-BoneRX implementation cohorts (see Table 2).

### 3.4. Satisfaction with BoneRx

Participants in the post-BoneRx implementation cohort were asked to complete questions regarding their experience receiving BoneRx (rating 1–10, higher = better). Mean scores were positive, and participants were satisfied with the bone health education they received (7.8 + 1.9), found it easy to understand (8.5 + 1.6), and felt that their knowledge about bone-related side effects of ADT treatment had increased (7.9 + 1.8).

## 4. Discussion

In this study, we compared two cohorts of men undergoing ADT for PCa before and after implementation of the BoneRx intervention. BoneRx is a simple tool that prompts the PCa specialist on guideline-adherent practice and provides clear recommendations and targeted education for the patient. Following the implementation of BoneRx, participants were more likely to have received a baseline BMD test and counselling about bone health from their HCPs. Furthermore, men in the post-implementation cohort reported engaging in more HBBs including vitamin D and calcium supplementation and moderate-to-vigorous physical activity. Feedback regarding the BoneRx intervention in those who received it was positive.

Encouragingly, we found that BoneRx was effective in increasing BMD tests at ADT initiation, which allows for accurate risk assessment and monitoring through follow-up assessments. Further, more men in the post-implementation cohort received counselling about bone from their cancer care team. Point-of-care reminders have been shown to be effective in increasing guideline adherent care and patient outcomes [47,48] and previous studies have found that providing reminders regarding BMD screening to HCPs and/or patients at risk of osteoporosis increases BMD measurement [60,61,62]. Tools, such as BoneRx, which include a reminder for providers and provide education to the target population, have the potential to increase BMD investigations, may reduce fracture rates [50], and highlight the need to consider multiple components and targets when developing tools and interventions to change care [63]. Patient understanding of the risk of OP due to ADT should be confirmed by encouraging questions and the opportunity to express any concerns [64]. This will contribute to an optimal patient-centred approach.

We also found that the implementation of BoneRx led to higher proportions of men engaging in important HBBs. Davison and colleagues found that an intervention for PCa survivors taking ADT that included a one-time nutrition class paired with a brochure increased calcium intake [65]. Interestingly, they also found that while men who were on ADT for less than 12 months increased their vitamin D supplement consumption after the intervention, men on ADT for more than 12 months did not [65]. This and other research suggest that patients may be more likely to undergo behaviour change closer to the time of diagnosis of their disease [66,67,68]. In the current study, all the participants had been on ADT for relatively short amounts of time (6 months or less).

Engagement in MVPA also increased after BoneRx implementation. However, it is important to note that only 37% of men were meeting the recommendation of 150 min of MVPA even after the BoneRx implementation. Barriers to exercise that PCa survivors face include lack of time, lack of willpower, having co-morbidities, increased age, and a lack of confidence following treatment [69,70,71]. Referrals to exercise specialists who can provide personalized exercise routines for patients may be able to help them overcome these barriers and build confidence to exercise after the completion of therapy [72].

Interestingly, despite the findings that HBBs increased following BoneRX implementation, PCa survivors’ OP knowledge or health beliefs were not different from those in the pre-implementation cohort group. Previous studies evaluating the effects of one-time interventions on OP knowledge in PCa survivors and the general elderly population have reported mixed results, with some studies finding improvements in knowledge scores and other studies finding no effects [51,62,73]. However, more comprehensive interventions consisting of multiple education classes have successfully improved OP knowledge of both individuals with the disease and the general population [74,75,76]. The finding that OP knowledge did not improve, yet HBBs did, does suggest that participants were better informed about HBBs. It is possible that the participants did not read the pamphlet and just relied on the healthy bone prescription, which provides very clear instructions. Research indicates that patients highly value the recommendations provided by their physicians and a physician’s advice is effective in encouraging patients to change their health behaviours [77,78]. In a previous pre-post study by our group [51], men receiving ADT were booked for BMD assessments and then sent personalized letters explaining their results and fracture risk assessment with an OP-related education booklet. While this intervention did not increase health motivation, it significantly increased OP knowledge and susceptibility scores [51]. Similarly, Sedlak and colleagues found that providing individual BMD results to post-menopausal women increased their perceived susceptibility to OP [79]. These findings suggest that incorporating personalized feedback from BMD tests into the BoneRx intervention may be helpful to increase PCa survivors’ knowledge and feelings of susceptibility.

There are limitations to this study that need to be considered. To begin, this was a quasi-experimental pre-post cohort group design. Not having a randomized design can introduce potential threats to internal validity. Further, this research was conducted at a single urban academic centre, thus, the results may have limited generalizability. The reliance on self-report for assessment of HBBs (e.g., exercise levels) introduces the possibility of reporting bias. Finally, long-term maintenance of HBBs or impact on important clinical outcomes such as fracture rates were not assessed and so, uncertainty remains as to whether this ultimately improves patient outcomes. Future studies should include a longer-term follow up to measure the maintenance of these behaviours and clinical outcomes. We also did not assess the long-term sustainability of using the BoneRx tool in the clinic.

## 5. Conclusions

BoneRx is a simple and acceptable strategy that can improve the care of PCa survivors receiving ADT. Our results suggest that this intervention can effectively remind physicians to provide guideline-concordant bone health care, such as ordering appropriate BMD tests and providing bone health counselling. Additionally, it can promote certain HBBs, such as vitamin D and calcium consumption and physical activity and has no demonstrable downsides or unintended consequences. Future iterations of this intervention may be explored to enhance patient outcomes, including providing multiple education sessions for expanding survivors’ OP knowledge, and/or providing personal BMD results for increasing perceived susceptibility to OP. Translation to other languages will also be important to increase health equity and patient-centred care.

## Figures and Tables

**Table 1:** Demographic and clinical variables of study participants.

	Pre-BoneRx Cohort (*n* = 143)	Post BoneRx Cohort (*n* = 149)	*p*-Value
**Age (mean/SD), y**	70.7 (±9.1)	70.4 (±8.5)	0.83
**Marital Status**			
Married/Common Law	102 (71%)	115 (77%)	
Single/Divorced/Widowed	42 (29%)	34 (23%)	0.22
**Language**			
English	127 (92%)	140 (94%)	
Other	11 (8%)	9 (6%)	0.52
**Employment**			
Employed (full, part, self)	42 (29%)	53 (35%)	
Retired	94 (65%)	92 (62%)	
Disability-leave, unemployed	8 (6%)	4 (3%)	0.28
**Education**			
High school	58 (41%)	39 (26%)	
College/University	57 (40%)	71 (48%)	
Post-graduate/Professional	27 (19%)	39 (26%)	**0.03**
**Treatment received ^a^**			
Hormone therapy	143 (100%)	149 (100%)	
Surgery	53 (39%)	61 (41%)	0.69
Radiotherapy	75 (55%)	93 (62%)	0.19
Chemotherapy	2 (2%)	5 (3%)	0.30
**Fracture Risk**			
Fracture after age 50 ^b^	15 (11%)	13 (9%)	0.62
Fall in last 12 months	31 (22%)	21 (14%)	**0.05**
Taken oral steroid medication	11 (8%)	20 (14%)	0.09

Numbers represent counts with percentages in parentheses unless otherwise indicated. ^a^ Percentages add up to >100% since participants could have received more than one treatment. ^b^ Rib, hip, wrist, or spine fracture at age > 50.

**Table 2:** Osteoporosis Knowledge Score and Osteoporosis Health Belief Scale Scores.

Outcome	Pre-Intervention (Mean ± sd)	Post-Intervention (Mean ± sd)	*p*-Value
**Osteoporosis Knowledge Score ^a^**	10.9 ± 3.6 (*n* = 142)	10.7 ± 3.8 (*n* = 147)	0.52
**Osteoporosis Health Belief Scale ^b^ (OHBS) Score**			
**Susceptibility**	17.1 ± 4.6 (*n* = 138)	16.7 ± 4.6 (*n* = 133)	0.41
**Seriousness**	17.5 ± 4.5 (*n* = 137)	17.4 ± 4.1 (*n* = 134)	0.79
**Motivation**	23.9 ± 4.0 (*n* = 136)	23.5 ± 3.2 (*n* = 141)	0.40
**Total**	58.6 ± 7.8 (*n* = 135)	57.4 ± 7.9 (*n* = 126)	0.25

^a^ Higher score indicates higher knowledge. ^b^ Higher score indicates better health beliefs (susceptibility, feelings of seriousness, health motivation).

## Data Availability

The datasets can be made available from the corresponding author upon reasonable request.
